# Effect of Carbon–Carbon Double Bond Content on the Final Properties of Stereolithography 3D-Printed Parts from Vegetable Oil-Based, Acrylated Resins

**DOI:** 10.1021/acsomega.5c06728

**Published:** 2025-10-21

**Authors:** Julius A. Adeyera, Julio A. Conti Silva, Kamran Kardel, Rafael L. Quirino

**Affiliations:** † Department of Manufacturing Engineering, 7604 Georgia Southern University, Statesboro, Georgia 30460, United States; ‡ Center for Advanced Materials Science, Biochemistry, Chemistry and Physics Department, Georgia Southern University, Statesboro, Georgia 30460, United States

## Abstract

The increase in the demand for additively manufactured parts and the drive toward a sustainable economy with less environmental pollution has triggered the need for renewable materials for use in 3D printing. This study shows how the final properties of stereolythography (SLA) 3D-printed parts are affected by the number of carbon–carbon double bonds in vegetable oils used to prepare biobased free radical resins. The free radical resins developed in this study contain a minimum of 95 wt % biobased content and are readily polymerizable using a commercial SLA 3D printer. Two distinct vegetable oils, namely soybean and linseed oils, were epoxidized and then acrylated. The epoxidized, acrylated oils and their 50:50 (wt/wt) blend were used to demonstrate the influence of carbon–carbon double bond content on the final properties of SLA 3D-printed parts. The synthetic strategy adopted was successful and showed promise, leading to printed materials with good thermo-mechanical properties. More specifically, it is demonstrated herein that the degree of unsaturation of the oil has a direct impact on the tensile strength of parts printed via stereolithography, and that final properties can be tuned by blending resins prepared from different oils. Ultimately, parts printed with linseed oil displayed better properties than those printed with soybean oil.

## Graphical abstract

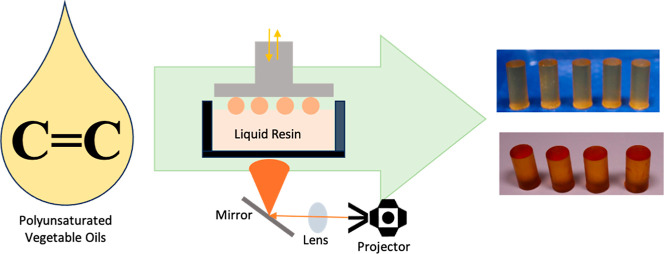

## 1 Introduction

The invention of 3D printing has transformed how products are designed and manufactured, enabling the rapid printing of complex and customized structures. There exist today various 3D printing technologies available for different applications, including stereolithography (SLA), Direct Light Processing (DLP), and Liquid Crystal Display (LCD). Collectively, these various 3D printing technologies are pushing the boundaries of manufacturing within healthcare, including bioprinting, tissue engineering, dentistry, surgical tools, drug delivery systems, prosthetics, medical devices,^1^ among many other applications. These technologies operate on a vat polymerization method, where a liquid photopolymer resin is polymerized through exposure to a light source (Figure 1).^2,3^ The first SLA 3D printer was patented by Charles Hull in 1986. Along with DLP, SLA is now one of the most common 3D printing methods due to its high accuracy and resolution.^4,5^ Following a layer-by-layer cure, SLA results in parts that are attached to a substrate or build plate.^2^ The photocurable resin is polymerized in this process by UV-induced polymerization (Figure 1), cross-linking all chemical monomers either continuously, or in layers to create 3D objects.^3,6^ The vat polymerization technology has evolved over the years, leading to significant improvements in the quality of printed parts.^7^ Higher resolution, smoother surfaces, stronger *z*-axis strength, and faster manufacturing are advantages of SLA over more traditional 3D-printing techniques, such as fused filament fabrication. However, the inflated cost of the high-resolution resin used in SLA and DLP limits their broader applicability.^8^

**Figure 1:**
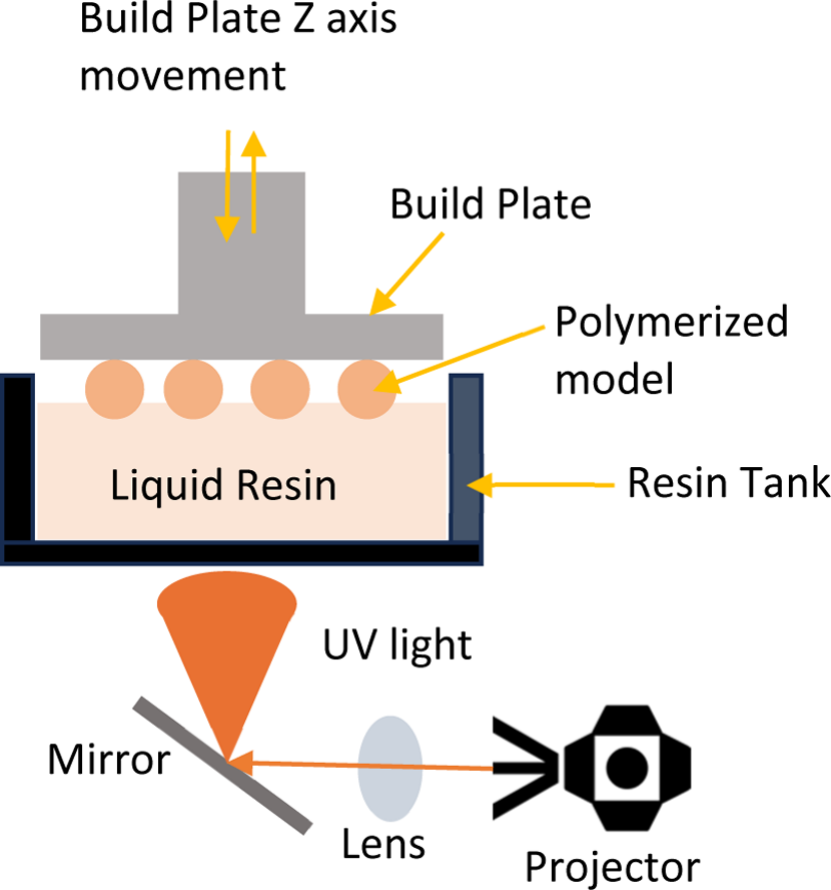
Schematic of vat polymerization method.

The rapid growth of 3D printing has led to an alarming increase in waste created by 3D printing plastics.^9^ Petroleum-based thermosets are often created by 3D printing processes, such as SLA and DLP, contributing to plastic pollution and accumulation.^10^ A significant obstacle to the widespread use of 3D printing is the need for environmentally safer resins.^11^ As a result, the scientific community has worked to create environmentally friendly substitutes for current conventional photopolymer resins in order to address the aforementioned issues.^12^ 3D printing technologies not only have the potential to reduce energy consumption and minimize waste, but can also be used to print a wide range of materials, including metals, ceramics, sand, food, polymers, and even living biological cells.^13^ Using biomaterials in 3D printers can reduce the environmental impact of additive manufacturing.^14^ Because biobased resins are prepared from biorenewable monomers, they represent a low carbon footprint process. For example, the soy plant captures CO_2_ from the atmosphere and produces, among other compounds, triglycerides (soybean oil) via photosynthesis. Therefore, the carbon backbone of soybean oil is constituted of Carbon that was originally found in the atmosphere, in the form of CO_2_. The oil extracted from soybeans can be used to prepare biobased resins for 3D-printing, like the ones described in this work. At the end-of-life of any parts prepared from a soybean oil-based resin, the eventual degradation of its polymeric network structure under specific environmental conditions restores CO_2_ to the atmosphere, where it came from in the first place. Therefore, globally, the increase in atmospheric CO_2_ levels is very limited. This concept is very clearly delineated in the literature.^15^ The design of such materials is penned as circular design and is in direct contrast with petroleum-based commodity plastics, for which stored carbon is eventually introduced into the atmosphere, resulting in a linear increase in atmospheric CO_2_ levels, with a negative environmental impact.^16^ Therefore, biologically derived materials are being produced as alternatives to fossil fuel-based materials.^17^

Biobased resins are typically made from acrylated vegetable oils or cellulose-derived polymers.^18^ Replacing petroleum-derived plastics and composite materials with biobased materials from inexpensive, environmentally friendly, natural, renewable resources would significantly impact the 3D printing industry.^19^ Previous studies have demonstrated that biobased materials can be used to create viable alternatives to petroleum-based polymers, such as methyl esters, epoxy resins, polyurethanes, polyesters, and vinyl ester resins.^20^

Vegetable oils have been used for the development of biobased resins for 3D printing.^21^ There are reports in the literature of resins prepared from rubber seed oil,^22^ waste cooking oil (consisting of a blend of soybean and canola oils),^23^ corn oil,^24^ and soybean oil.^25^ A library of soybean oil-based resins was synthesized from the mixture of soybean oil and different combinations of methacrylate oligomers, biobased diluents, and a photo initiator. Vegetable oil derivatives have been explored as a main ingredient for UV printable inks by conducting a thorough analysis of different formulations based on acrylated, epoxidized soybean oil (AESO), or mixed reactive diluents.^26^ Very recently, the preparation of a methacrylated microalgal oil-based resin for SLA 3D printing was successfully demonstrated,^27^ as well as the preparation of photocurable resins with high acrylate functionality by the incorporation of itaconic anhydride-pentaerythritol triacrylate adducts onto epoxidized soybean oil.^28^ Miao S. et al. used AESO to print smart biomedical scaffolds.^29^ Several studies have focused on developing biobased resins for SLA printing under optimized curing parameters, like the 2D and 3D photolithographic bioprinting of soy-based scaffolds for cell-cultured meat using vat polymerization.^30^ Furthermore, the commercial resin Bio Soya1 (Clear, P11892/2I, 3Dresyns) was created from soybean oil for monochrome LCD printing with a *z*-resolution thickness of 50–100 μm. In conclusion, using biobased resins in SLA printing is a potential approach to reduce the negative environmental effects of 3D printing using petroleum-based products. Several studies have shown that bioresins can have excellent mechanical and print properties. However, additional research is required to enhance the mechanical properties of biobased resins for SLA printing and to optimize the curing process.

Although the use of acrylated oils in 3D-printing has been covered in the literature with interesting reviews recently published,^28,31^ there is a persistent gap in direct comparison of performance of different oils based on their degree of unsaturation. The qualitative comparison of resins prepared from different oils traditionally relies on data from different reports, using resins prepared by different groups and printed using different printers, sometimes under different conditions. When comparing these different systems, there is little emphasis in explaining the properties obtained from a molecular point of view, and although critical, the degree of unsaturation of oils is often overlooked. Therefore, by carrying a systematic study, the number of varying parameters can be limited and a relationship between the degree of unsaturation of vegetable oils and the final properties of printed parts can be established. Moreover, this study aims at demonstrating that it is possible to effect changes in properties by combining oils with distinct degrees of unsaturation at different proportions.

In this work, the synthesis of acrylated, epoxidized linseed oil (AELO) and AESO resins and their blend will be discussed, as well as their printability, resolution, and characterization of printed parts. More specifically, this paper investigates the curing time, mechanical properties, and chemical composition of LCD 3D-printed, acrylated vegetable oils. The work presented herein provides a systematic evaluation of the effect of the degree of unsaturation of two vegetable oils of interest, namely soybean and linseed oils, on the thermo-mechanical properties of 3D-printed parts prepared from their acrylated, epoxidized resins. Soybean oil, due to its abundance and low cost in large markets, such as the US, has received considerable attention as a biorenewable resource for 3D printing resins, while more unsaturated oils, such as linseed oil, can potentially lead to resins and printed parts with better properties. The findings of this work provide more insights on critical considerations for the development and characterization of specimens made from AELO, AESO, and their blend as biobased free radical resins via LCD 3D printing.

Because it has been long established that synthetically adding acrylate groups to epoxidized vegetable oils increases their reactivity toward free radicals,^32–38^ that strategy has been borrowed here to prepare fast-curing resins for SLA 3D printing with simple chemistry. It is important to stress that the novelty of this work lies in the systematic evaluation of the impact of the carbon–carbon double bond content of vegetable oils on the final properties of printed parts, rather than on the chemical modification of the oils. Indeed, there is a robust body of work about the epoxidation of vegetable oils,^39^ their subsequent acrylation,^40^ and the use of this strategy for the preparation of vegetable oil-based resins from soybean,^32–35,38^ olive,^36^ and waste cooking oils for SLA 3D printing,^37,38^ making this an ideal and simple synthetic strategy for the study of the effect of carbon–carbon double bonds on the final properties of printed parts. However, the use of epoxidized linseed oil for the preparation of SLA 3D printing resins has relied primarily on the direct cationic polymerization of the oxirane group via a ring-opening reaction,^39,41^ in contrast with the free radical polymerization of acrylate groups adopted in the current work. Most importantly, in the available literature, the fundamentally important correlation between the carbon–carbon double bond content and the final properties of printed parts is not clearly contemplated, setting the work presented herein as a critical step in understanding the cure of vegetable oil-based resins and allowing for the tuning of final properties via blending of resins prepared with distinct oils for their real-world applications.

## 2 Materials and Methods

### 2.1 Materials

Linseed oil (LO) and soybean oil were purchased from MP Biomedicals (Solon, OH, USA) and US Organic Group Corp, respectively. Hydrogen peroxide (30.0 wt % aqueous solution), acrylic acid, toluene, acetic acid, sulfuric acid, anhydrous sodium sulfate (Na_2_SO_4_), ethyl acetate, dichloromethane, and hydroquinone were purchased from Fisher Scientific (Ward Hill, MA, USA). The photo initiator phenyl bis­(2,4,6-trimethylbenzoyl) phosphine oxide (Irgacure 819) was purchased from TCI America (Portland, OR, USA). Deuterated chloroform was procured from Cambridge Isotope Laboratories (Tewksbury, MA, USA). *Iso*-propyl alcohol (99%) was obtained from Florida Laboratories, Inc. (Fort Lauderdale, FL, USA) and the commercial resin Bio Soya1 was purchased from 3Dresyns (Barcelona, Spain). All reagents were used as supplied without any further purification.

### 2.2 Determination of C=C Content in Vegetable Oils

In order to estimate the average number of C=C bonds in each oil, integration of olefinic and alpha methylene proton signals in the corresponding proton nuclear magnetic resonance (^1^H NMR) spectra of the oils was used. Since each unsaturation corresponds to two protons, the number of carbon–carbon double bonds per oil can be estimated by eq 1

1 $$N=\frac{3A_{j}}{A_{f}}$$

where *N* is the average number of C=C bonds per oil, *A*_*j*_ is the integrated area of the olefinic proton (−CH=CH−) signals (∼5.2 ppm—signal “j” in Figure 2), and *A*_f_ is the integrated area of the signal associated with the protons bonded to carbons alpha to ester groups (∼2.2 ppm—signal “f” in Figure 2). It was determined that LO had an average of 5.8C=C per molecule, whereas SO had 4.1 C=C per molecule.

**Figure 2:**
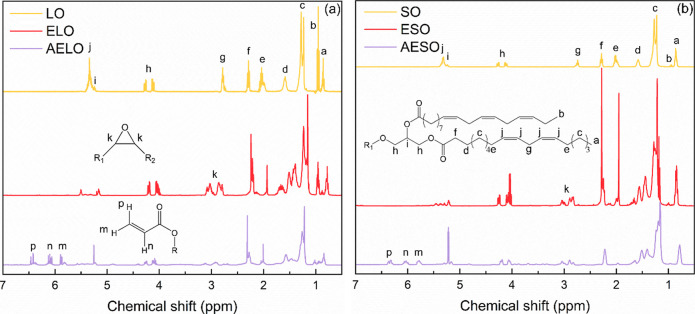
^1^H NMR spectra of (a) LO, ELO, and AELO, and (b) SO, ESO, AESO, with attributions to the most relevant signals.

### 2.3 Synthesis of Epoxidized Vegetable Oils

The procedure for the epoxidation of soybean and linseed oils was adapted from the literature.^42^ For the epoxidation reaction, 200 g of a vegetable oil, namely LO or SO, and acetic acid, in the proportion of 0.5 equiv of acid per equivalent of C=C in the oil (corresponding to 41.28 g of acetic acid for the reaction with LO, and 26.09 g of acetic acid for the reaction with SO), were added to an Erlenmeyer flask alongside 100 mL of toluene and 3 wt % of a 1.0 M sulfuric acid aqueous solution (relative to the mass of oil used). Then, 30 wt % hydrogen peroxide aqueous solution was added dropwise to the mixture in a proportion of 1.5 equiv of peroxide per equivalent of C=C of the oil used. The mixture was kept under agitation at 70 °C for 3 h. At the end of the reaction, the mixture was cooled down to room temperature and dissolved in 100 mL of ethyl acetate. The contents were transferred to a separatory funnel and washed three times with 200 mL of water at 60–75 °C to bring the pH to neutral. The organic layer was isolated and dried by adding approximately 36 g of anhydrous sodium sulfate. The epoxidized oil was recovered after vacuum filtration and the solvent was evaporated under reduced pressure. The products, namely epoxidized linseed oil (ELO), and epoxidized soybean oil (ESO), were characterized by Fourier Transform Infre-red (FTIR) and ^1^H NMR spectroscopies, following a previously published protocol.^42^

### 2.4 Acrylation of ELO and ESO

The procedure for the acrylation of ELO and ESO was adapted from the literature.^42^ For the acrylation of epoxidized vegetable oils, 200 g of either ELO, or ESO, acrylic acid (in the proportion of 1 equiv of acrylic acid per equivalent of C=C in the original oil, corresponding to 86.9 g of acrylic acid for 200 g of ELO, and 57.6 g of acrylic acid for 200 g of ESO), 1 wt % of triethylamine (with respect to the total weight of reagents), and 0.2 wt % of hydroquinone (with respect to the total weight of reagents) were added to a round-bottom flask and kept under agitation for 5 h at 75 °C.

### 2.5 Product Purification

The product was then dissolved in 100 mL of dichloromethane, transferred to a separatory funnel, and extracted three times with 300 mL of water at 60–75 °C to ensure complete removal of unreacted triethylamine, acrylic acid, and hydroquinone. The organic layer containing the acrylated oil was then isolated and dried with approximately 39 g of calcium chloride to remove traces of water. Calcium chloride was removed from the products by vacuum filtration and dichloromethane was evaporated under reduced pressure, leaving only the acrylated oil as the product. The final products, namely acrylated epoxidized linseed oil (AELO) and acrylated epoxidized soybean oil (AESO), were characterized by FTIR and ^1^H NMR spectroscopies.

### 2.6 Preparation of Photocurable Resins

To prepare the photocurable resins used in this work, 1 wt % of photo initiator Irgacure 819 (with respect to the total weight of the acrylated, epoxidized oils used) was mixed with AELO, AESO, or a 50:50 (wt/wt) blend of AELO and AESO, and sonicated in a sonication bath for approximately 20 min at 55 °C. The resin compositions tested are detailed in Table 1.

**Table 1:** Photocurable Resin Compositions Tested in This Work

sample name	AELO (wt %)	AESO (wt %)
AELO	100	0
75(AELO):25(AESO)	75	25
(AELO):(AESO)	50	50
AESO	0	100

### 2.7 3D Printing Process

An Elegoo Saturn 2 3D LCD printer (Shenzhen, China) was used to 3D-print all biobased resins. The printer has a monochrome LCD with a resolution of 8000 LEDs/10 in. Each LED provides UV at a wavelength of 405 nm. The printer has a build volume of 219 mm × 123 mm × 250 mm and a *Z* axis accuracy of 0.01–0.20 mm. Digital models were designed using the SolidWorks software and exported as a Standard Tessellation Language (STL) file. Elegoo Chitubox slicer software was used to process the STL files considering mode, support, orientation, and lift speed. The G-code was automatically generated by the Chitubox software. The STL files were then sent to the Elegoo 3D printer for printing. The printing parameters used were 40 s of exposure time and a layer thickness of 50.0 μm. The same print parameters (Table 2) were used for printing all resins. The printing process was conducted at 23–25 °C.

**Table 2:** Printing Parameters Used for Printing all Samples

parameters	values
layer thickness	50 μm
laser wavelength	405 nm
normal exposure time	40 s
bottom layer count	5
rest time after retract	0.5 s

In a typical print, the vat is filled with the photocurable resin. Each layer is imprinted on the previous layer as the build plate moves upward (Figure 1). Figure 3 shows printed specimens prepared for compression testing still attached to the build plate. After the printing process is completed, the printed specimens are removed from the build plate and the sample is submitted to a postcure step. Finally, the samples are washed with a mixture of 70:30 (vol %, *iso*-propyl alcohol/distilled water) and dried under ambient conditions.

**Figure 3:**
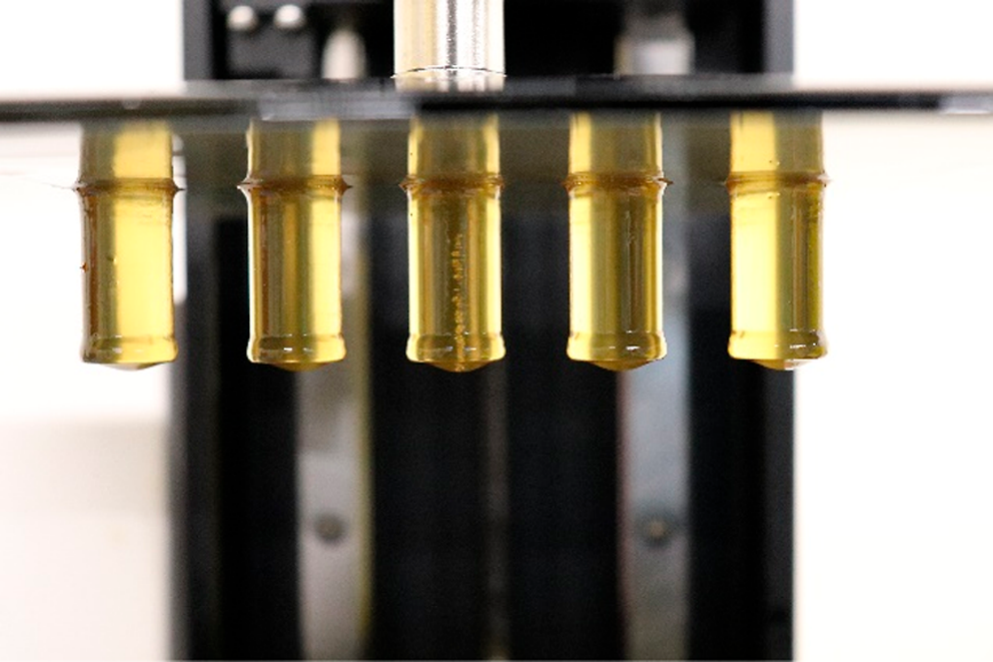
AESO 3D-printed compression test specimens still attached to the build plate.

### 2.8 Characterization

^1^H NMR spectra were obtained with an Agilent MR400DD2 spectrometer (Santa Clara, CA), operating at 400 MHz. The samples analyzed were dissolved in deuterated chloroform. FTIR spectra were collected using a Thermo Nicolet iS10 FTIR spectrometer (Thermo Scientific, Waltham, MA) equipped with an attenuated total reflectance (ATR) accessory. The spectra were obtained in the 650–4000 cm^–1^ spectral region, with 32 scans, and 4 cm^–1^ resolution.

Viscosity measurements were performed on a Discovery HR-2 Hybrid Rheometer (TA Instruments, New Castle, DE), using parallel plates geometry. All measurements were performed isothermally at 25 °C. The shear rate increased stepwise from 1 to 1000 s^–1^ and 16 data points were collected. The values reported correspond to the average of measurements performed. DSC experiments were conducted with a Discovery Series Q250 DSC instrument (TA Instruments, New Castle, DE). Tests were run in a N_2_ atmosphere using Tzero hermetically sealed aluminum pans with a sample size of approximately 10 mg. Each sample was heated from 25 to 275 °C at 10 °C/min. Dynamic Mechanical Analysis (DMA) was performed on printed specimens with dimensions of 20 mm × 10 mm × 2 mm (length × width × thickness) using a Q800 DMA (TA Instruments, New Castle, DE) with a three-point bending fixture. A limitation was observed with respect to the lower temperature during the DMA experiments, with the samples breaking during the initial cooling at approximately −20 °C. A decision was therefore made to start all DMA measurements at −15 °C to avoid the premature failure of the samples. All samples were then initially cooled to −15 °C and subsequently heated to 150 °C at a rate of 3 °C/min in iso-strain mode at a frequency of 1 Hz and amplitude of 14 μm.

A MTS Criterion model 43 Universal Testing Machine (Eden Prairie, MN, USA) with a crosshead speed of 0.1 mm/s was used for compression and tensile tests. Compression testing was performed according to ASTM D695 using a load cell of 30 KN. Cylindrical specimens with 1.0″ height and 0.5″ diameter were printed perpendicular to the build direction for the analysis. A total of five specimens were tested for each sample and the results reported represent the average of those measurements. Tensile testing was performed according to ASTM D638 using a load cell of 10 KN. Type V specimens with 2.87 mm thickness, 3.40 mm width, and 9.53 mm length were printed perpendicular to the build direction for tensile testing. A total of five specimens were tested for each sample and the results reported represent the average of those measurements. Hardness testing was conducted with a hand-held Shore D Durometer (Gain Express Holdings Ltd., Kowloon, Hong Kong) on 2″ × 2″ × 0.25″ specimens printed perpendicular to the build direction in accordance with ASTM D2240. For each sample, two specimens were printed and three measurements were performed in each specimen, totaling 6 measurements per sample. The results reported herein for each sample represent the average of those measurements.

## 3 Results and Discussion

### 3.1 Characterization of Crude Resins

Both oils, their epoxidized products, and acrylated epoxidized resins were characterized by FTIR and ^1^H NMR spectroscopies. Both LO and SO presented FTIR signals centered at 2923 cm^–1^ and 2853 cm^–1^, related to H–C­(sp^3^) stretching vibrations, and at 3010 cm^–1^, resulting from H–C­(sp^2^) stretching vibrations, due to the presence of unsaturations in both oils’ structures (Figure 4). The signal centered at approximately 1750 cm^–1^, related to C=O stretching vibrations from ester groups in the triglyceride is also noticeable in both oils’ spectra (Figure 4).^43^ After the epoxidation reaction, the appearance of the characteristic C–O–C stretching signals of the oxirane ring at approximately 846 cm^–1^ and 821 cm^–1^ indicate the successful epoxidation of the oils (Figure 4).^44^ The residual signal at 3010 cm^–1^ in ELO and ESO suggests that not all carbon–carbon double bonds are epoxidized (Figure 4). The ring-opening of oxirane groups in the epoxidized oils with acrylic acid also resulted in new signals, such as the O–H stretching centered at ∼ 3500 cm^–1^ and the signals centered at 1636 cm^–1^ and 810 cm^–1^ (Figure 4), related to C=C stretching and C=C out-of-plane bending, respectively, of the vinyl moieties of the acrylate groups.^45^ Indeed, the OH stretch observed on Figure 4 for AELO and AESO is attributed to the hydroxyl group resulting from the ring-opening reaction of the oxirane ring during acrylation. Residual signals at 846 cm^–1^ in AELO and AESO indicate that not all epoxide groups are converted (Figure 4). A reaction scheme for the epoxidation of vegetable oils followed by their acrylation is depicted in Figure 5.

**Figure 4:**
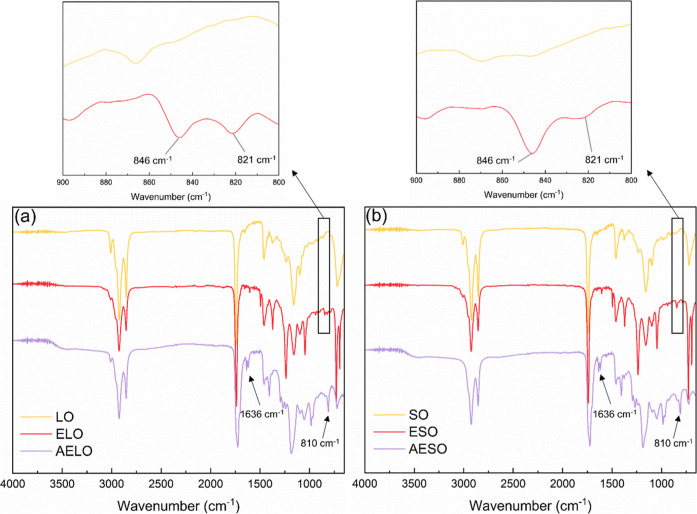
FTIR spectra of (a) LO, ELO, and AELO, and (b) SO, ESO, and AESO. The inserts correspond to a zoom of the 900–800 cm^–1^ spectral region.

**Figure 5:**
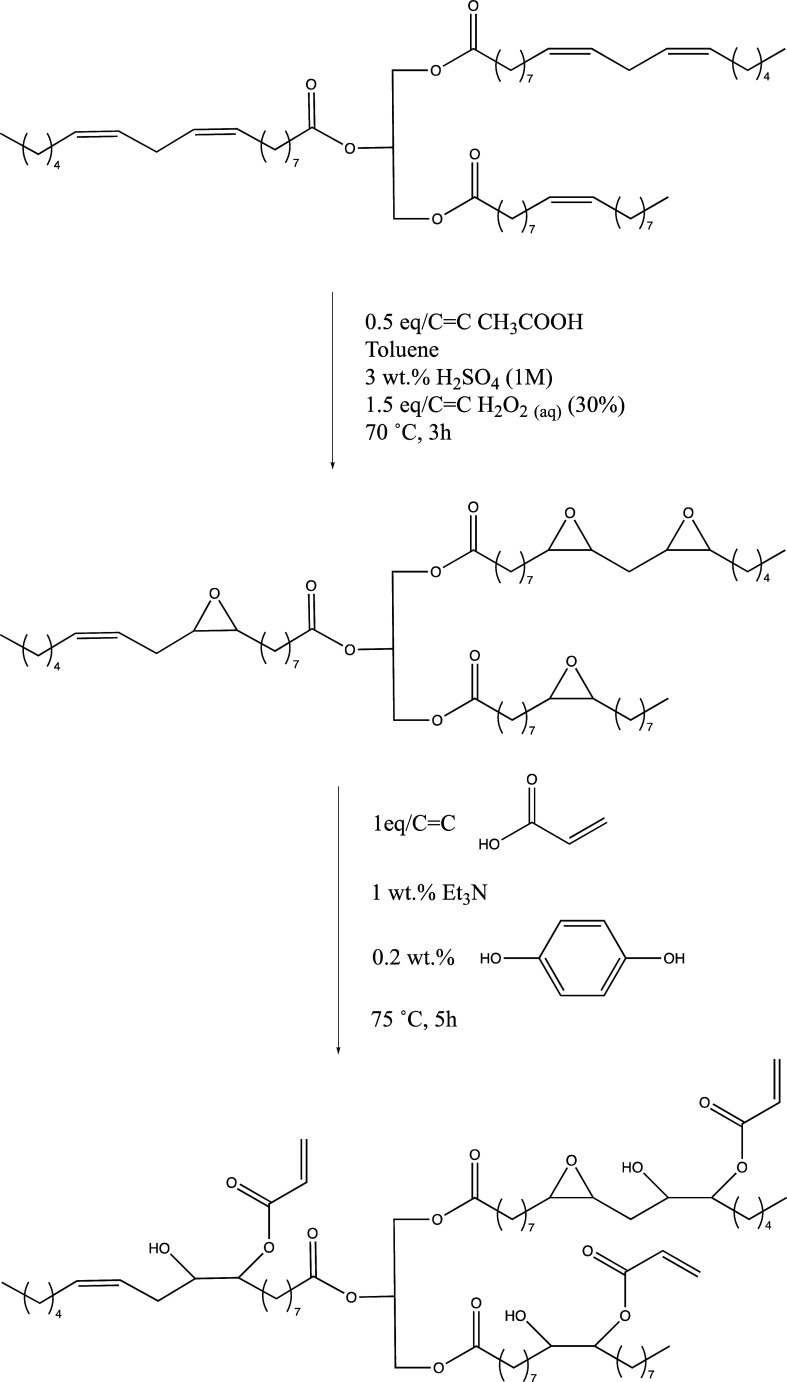
Overall reaction scheme for the preparation of acrylated, epoxidized vegetable oils using a model, polyunsaturated triglyceride.

^1^H NMR spectroscopy (Figure 2) confirmed the presence of carbon–carbon double bonds in both oils’ structures with characteristic chemical shifts of olefinic protons (−CH=CH−) observed at approximately 5.31 ppm for SO, and 5.33 ppm for LO. The characteristic signal of *bis*-allylic protons (=CH–CH_2_–CH=) can be observed at 2.74 ppm for SO, and 2.78 ppm for LO (Figure 2). Integration of the ^1^H NMR signals reveals that LO has approximately 5.8 carbon–carbon double bonds per triglyceride, whereas SO has 4.1 carbon–carbon double bonds per triglyceride. The triglyceride structure of the oils is confirmed by the signals at 4.14 and 4.26 ppm (Figure 2), characteristic of glycerol protons. The presence of these signals in the spectra of all products indicate that after epoxidation and acrylation, the triglyceride backbone was preserved. Both epoxidized products presented a new signal at 3.04 ppm (Figure 2), corresponding to oxirane protons (−CH–O–CH−), while the olefinic and bis-allylic proton signals became less intense. Integration of the olefinic proton signals indicate that both ELO and ESO present only one carbon–carbon double bond per triglyceride.^46^ Acrylation resulted in the appearance of signals at 5.70–6.49 ppm (Figure 2), assigned to the three protons of acrylate esters, suggesting the successful introduction of acrylate moieties to the epoxidized oils. The presence of oxirane protons in the spectra of AELO and AESO (Figure 2) indicate that acrylation did not proceed until full conversion, which is in line with previous reports.^47^ Integration of the acrylate peaks suggest that AELO presents an average of 3.07 acrylate groups per triglyceride, representing a conversion rate of 53% of carbon–carbon double bonds into acrylate groups, whereas AESO has 2.40 acrylate groups per triglyceride, representing a conversion rate of 56% of carbon–carbon double bonds into acrylate groups. It is worth noting that no signals were observed above 7 ppm in the ^1^H NMR spectra of AELO and AESO, confirming the absence of carboxylic acids in the products.

As well reported in the literature, many formulations of acrylated systems include reactive diluents that help reduce the viscosity of the resin before and during printing, while being incorporated into the final polymer network and contributing to the final properties of printed parts.^26^ The final step in the synthesis of the acrylated resins reported herein involves solvent removal under reduced pressure. No problems with the viscosity of these resins were experienced during printing, therefore diluents were not needed here. To better illustrate this, the viscosity of the resin prepared from linseed oil (AELO) was measured and contrasted to a commercial biobased resin (Bio Soya1). While AELO exhibits a viscosity of 22 mPa s at 25 °C, Bio Soya1 exhibits a viscosity of 156.5 mPa s, both resulting in fine printed parts, with no indication of viscosity problems.

### 3.2 Postcure Analysis

FTIR analysis of 3D-printed resins (Figure 6a) shows that, for AESO and the (AELO):(AESO) blend, the characteristic acrylate signals (1636 cm^–1^ and 810 cm^–1^), originally present in the crude resins (Figure 4), disappeared, suggesting complete polymerization of acrylate groups for those samples. For AELO, those characteristic signals are still present in the spectrum of the printed sample, indicating the need for postcuring. The low intensity of these signals suggests polymerization of most acrylate groups. Unfortunately, quantification based on FTIR is not satisfactorily reliable for an accurate calculation of acrylate conversion yield. Additionally, the cross-linked nature of the polymerized resin makes it insoluble in common deuterated organic solvents for NMR analysis. AELO has more acrylate groups per triglyceride than AESO, leading to a higher cross-link density after polymerization. During the 3D-printing process, both resins and their blend yield solid pieces that are able to be fully recovered. It is likely that, despite reaching a gel point, some of AELO’s acrylate groups still remained unpolymerized. The early gel point achieved hinders chain mobility, compromising full polymerization. Additionally, AELO was darker in color compared to AESO (Figure 6b), which can impact the penetration depth of light, consequently affecting full polymerization across each layer. In the literature, it has been observed that acrylated resins of canola, sunflower, and soybean oils (all very similar in color and containing similar degrees of unsaturation) possessed comparable penetration depths, while the penetration depth for a considerably darker acrylated sesame oil resin exhibited a lower penetration depth.^48^

**Figure 6:**
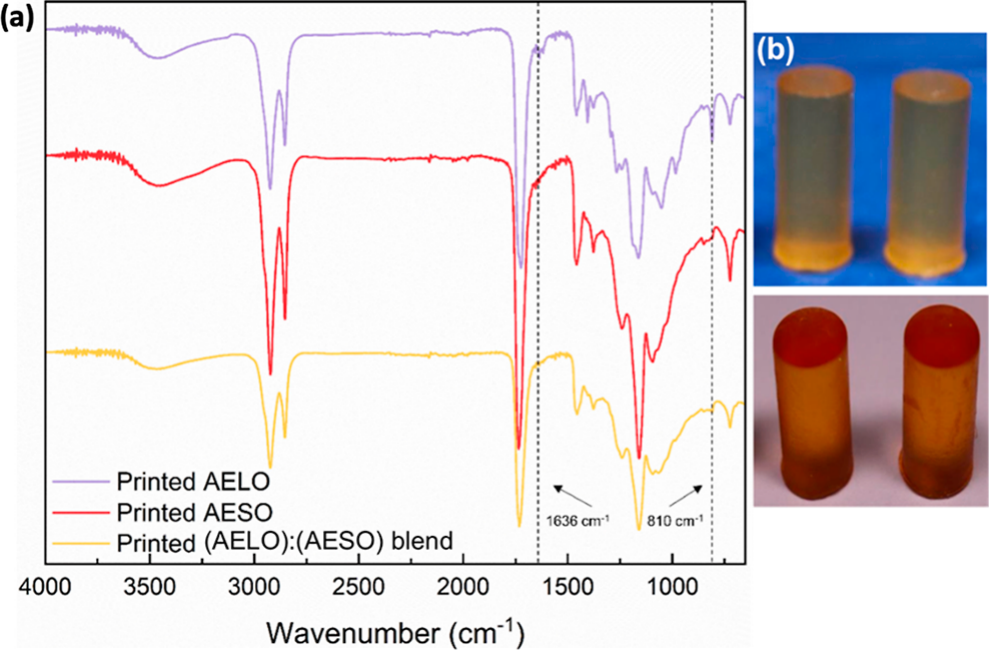
(a) FTIR spectra of printed resins. (b) 3D-printed compression test specimens of AESO (top) and AELO (bottom).

In order to ensure polymerization completion after printing, it is customary to perform a postcure of printed parts. Because the penetration depth of the UV radiation is significantly reduced after initial printing, making it impossible to postcure the core of the parts printed, the postcure was carried out in an oven. In this case, the entire part (including the core) attains the desired temperature, ensuring full cure of the part. In preliminary tests (not displayed here), samples postcured under UV for up to 20 min still exhibited a significant DSC exothermic peak, indicating that the cure was not completed.

To determine optimum postcure conditions, three AELO test specimens with dimensions of 20 mm × 10 mm × 2 mm (length × width × thickness) were printed and subjected to a postcure at 130 °C for different times (Figure 7). The temperature of 130 °C was arbitrarily chosen taking into consideration temperatures typically used for the thermal free radical polymerization of carbon–carbon double bonds in vegetable oil-based thermosetting resins.^49^ It can be seen in Figure 7 that after 14 min of postcure at 130 °C, the sample exhibits a large exothermic peak centered at approximately 180 °C, associated with the polymerization of unreacted carbon–carbon double bonds in the sample. These unreacted carbon–carbon double bonds can be either from unreacted acrylate groups, or unreacted unsaturations from the oil backbone itself. After 18 min of postcure, a much smaller exothermic peak is observed (Figure 7), and after 30 min no exothermic peaks are observed for the sample, indicating that the polymerization is complete. Due to the cross-linked nature of the cured samples, they are insoluble in common deuterated organic solvents for NMR analysis, therefore the relative extent of carbon–carbon double bond polymerization is inferred from the intensity of the exothermic peak on the DSC curve (Figure 7). These results highlight the need for a postcure step after printing, without which the printed parts would not be completely polymerized, potentially leading to less-than-ideal materials that are susceptible to oxidation and structural changes over time. Due to the lower number of carbon–carbon double bonds in soybean oil (4.1 C=C/triglyceride) with respect to linseed oil (5.8 C=C/triglyceride), the postcure conditions adopted for AELO are expected to fully polymerize AESO. Therefore, all AESO and AELO printed samples were subjected to a postcure of 30 min at 130 °C for the remainder of the work presented herein.

**Figure 7:**
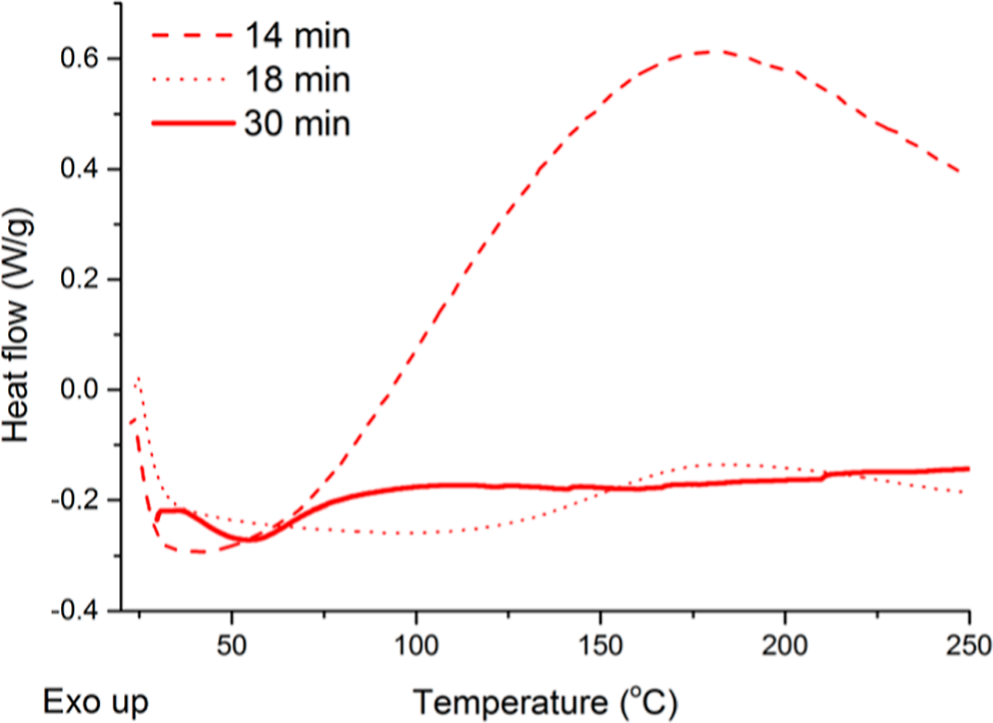
DSC curves of AELO 3D-printed specimens subjected to different postcure times at 130 °C.

### 3.3 Characterization of Printed Resins

DMA results of the printed samples are presented on Table 3 and Figure 8. Table 3 shows the storage modulus (*E*′) of the samples at two distinct temperatures, namely, at 25 °C, representing how the material behaves at room temperature, and at 50 °C above the glass transition temperature (*T*_g_), representing the behavior of the materials at the rubbery state. The *T*_g_’s were determined based on the temperatures of maximum tan delta values (Figure 8). As seen in Table 3, all *T*_g_’s occur between 17 °C and 48 °C. The temperature range selected for the DMA experiments (from −15 °C to 150 °C) encompasses these three critical points, namely (1) the *T*_g_, (2) room temperature (reflecting properties for most real-world applications), and (3) the rubbery plateau (>75 °C) for all samples investigated. All DMA curves follow a classical behavior profile for thermosetting resins, as expected, with the storage modulus decreasing as the temperature increased, until it plateaus, indicating the sample attained the rubbery state. The DMA results also demonstrate a clear trend, in which printed AELO had a higher *T*_g_ and storage modulus at both glassy and rubbery states when compared to printed AESO. This result is associated with the higher number of C=C/triglyceride in LO in comparison to SO. A similar trend has been reported in the literature for the *T*_g_ of polymerized acrylated linseed (6.6 °C), grapeseed (−1.7 °C), and rapeseed (−3.6 °C) oils, matching their respective degree of unsaturations (5.99 C=C/triglyceride, 4.56 C=C/triglyceride, and 3.74 C=C/triglyceride).^50^ Similarly, the addition of cross-linkers to photopolymerized AESO increased the *T*_g_ and the storage modulus of the material.^26^

**Table 3:** DMA Results, Including Storage Modulus (*E*′) at 25 °C and at the Rubbery State (*T*_g_ + 50 °C), Glass Transition Temperatures (*T*_g_), and Crosslink Density (ε) of Printed AELO Resin, AESO Resin, and Their Blend

sample	*E*’ at 25 °C (MPa)	*T*_g_ (degC)	*E*’ at *T*_g_ + 50 °C(MPa)	ε (10^–7^ mol/cm^3^)
AELO	176 ± 60	48 ± 9	5 ± 2	5.4
(AELO):(AESO) blend	51 ± 14	17 ± 3	6 ± 2	7.1
AESO	13 ± 4	18 ± 7	1 ± 1	1.2

**Figure 8:**
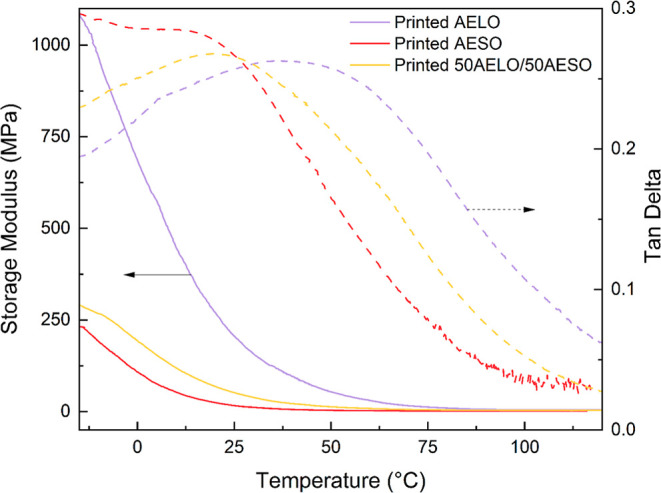
DMA curves of the 3D printed resins representing storage modulus (*E*′) and tan delta as functions of temperature.

The broad fatty acid composition of vegetable oils is notoriously responsible for resulting in polymers with heterogeneous network structures. It is not uncommon to observe very broad tan delta peaks for vegetable oil-based thermosets, as frequently reported in the literature.^51^ It is also worth noting that the correlation between the initial degree of unsaturation of the oils and the final properties of the printed parts is directly associated with the acrylate functionality added through the synthesis performed. Indeed, a greater number of carbon–carbon double bonds per triglyceride will naturally result in a monomer with more acrylate groups, and should logically result in a polymer with more cross-links and higher mechanical properties. Similar trends had been suggested in the literature,^52^ but neither a direct and systematic evaluation of 3D-printed parts had been contemplated, nor blends of oils with distinct degrees of unsaturation had been assessed.

Cross-link densities (ε) were calculated based on the rubbery elasticity theory from the average storage modulus at *T*_g_ + 50 °C reported in Table 3. According to Flory’s Rubber Elasticity Theory, the molecular weight between cross-links (*M*_c_) is defined by eq 2.^53^

2 $$M_{c}=3RT\rho /E^{\prime }$$

where ρ is the polymer density, *E*′ is the storage modulus at *T*_g_ + 50 °C, R is the gas constant (8.314 m^3^ Pa K^–1^ mol^–1^), and T is the absolute temperature. By defining the cross-link density (ε) as ρ/*M*_c_,

Equation 3 is obtained. Equation 3 was then used to calculate the cross-link densities reported in Table 3.

3 $$\varepsilon =E^{\prime }/3RT$$

It is interesting to observe that, for temperatures below approximately 60 °C, the storage modulus of the (AELO):(AESO) blend is intermediate between pure AELO and pure AESO resins, as expected based on the number of unsaturations per triglyceride in LO and SO. Surprisingly, the *T*_g_ of the printed (AELO):(AESO) blend is very similar to that of AESO, and in the rubbery plateau (>98 °C) the (AELO):(AESO) blend exhibits a higher storage modulus than both AESO and AELO. This results in a higher calculated cross-link density for the (AELO):(AESO) blend than for either of the pure resins. It is possible that during polymerization of the blend, the less reactive AESO works initially as a diluent, improving chain mobility to ensure an overall higher conversion/reaction of carbon–carbon double bonds, resulting in a higher cross-link density. Without the diluent effect, pure AELO gels faster (as explained during the discussion of Figure 6), resulting in limited chain mobility, compromising full reaction of carbon–carbon double bonds, and negatively affecting the final cross-link density. For AESO, the lower initial number of C=C/triglyceride impacts both storage modulus and cross-link density.

The results obtained for cross-link densities of the printed resins are significantly lower than those obtained for tung oil directly polymerized with other comonomers (25.9 × 10^–7^ mol/cm^3^) and in the range of copolymers of single fatty acids (5.4 × 10^–7^ mol/cm^3^).^54^ This is in line with the conversion yield of C=C in acrylate groups as explained in the discussion of Figure 2 earlier in the text. A similar behavior had been observed for vegetable oil-based thiol–ene and thiol-epoxy resins, in which the addition of ELO to a mixture of AESO and thiol produced polymers with higher cross-link density, but the *T*_g_ remained analogous to the polymers produced in the absence of ELO.^55^

The ability to adjust and control the mechanical properties of materials is one of the advantages of 3D printing systems and is crucial in the development of objects intended for engineering and biomedical applications.^1^ The tensile tests performed generated stress–strain curves, from which the Young’s Modulus (*E*) was determined from the slope of the tangent in the initial 10% of the curve. Additionally, tensile strength and strain at break were also obtained from the tests. A graph of representative stress–strain curves for AELO, AESO, and their blend is presented in Figure 9, while comprehensive results are summarized in Table 4. The results show AELO having the highest tensile strength (14.94 ± 1.28 MPa), followed by the (AESO):(AELO) blend (4.39 ± 0.26 MPa), and AESO having the lowest tensile strength (2.21 ± 0.33 - Figure 9 and Table 4). These results are in line with the storage modulus at 25 °C reported in Table 3 and discussed earlier in the text. As previously explained, the higher degree of unsaturation of LO results in more acrylate groups, and consequently in a higher cross-link density, which is reflected in higher mechanical properties. For context, the Young’s modulus of another AESO resin, prepared by a different group using a different synthetic approach, had been previously reported in the literature as 1.43 ± 0.37 MPa, while the tensile strength was 3.46 ± 0.25 MPa and the strain at break was 3.44 ± 0.46%.^28^

**Figure 9:**
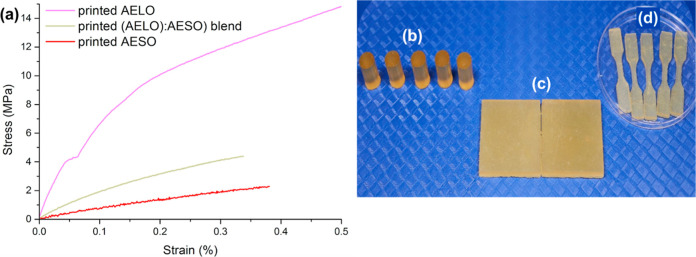
(a) Representative stress–strain curves from the tensile testing of AESO, (AELO):(AESO) blend, and AELO. (b) AELO specimens used for compression testing. (c) AESO specimens used for hardness testing. (d) AESO specimens used for tensile testing.

**Table 4:** Tensile and Compressive Test Results

sample	young’s modulus (MPa)	tensile strength (MPa)	strain at break (%)	compressive modulus (Mpa)	compressive strength (MPa)	hardness (0–100)
AELO	77.02 ± 8.52	14.94 ± 1.28	0.50 ± 0.10	722.92 ± 86.36	51.60 ± 5.37	57.3 ± 1.52
75(AESO):25(AELO) blend^a^				743.19 ± 2.46	46.27 ± 7.56	
(AESO):(AELO) blend	25.10 ± 1.76	4.39 ± 0.26	0.28 ± 0.07	681.71 ± 27.03	41.95 ± 1.48	42.7 ± 0.29
AESO	8.58 ± 0.71	2.21 ± 0.33	0.38 ± 0.05	102.05 ± 3.75	9.80 ± 0.28	22.8 ± 0.76

a A limited amount of this specific blend was prepared to confirm the overall trends observed for compression testing.

The tensile tests of AELO, AESO, and their blends revealed distinct mechanical properties that could be related to the underlying chemistry, focusing on copolymerization and performance characteristics, as well as the influence of unsaturated vegetable oils. The Young’s moduli (*E*) for AELO, the (AELO):(AESO) blend, and AESO were 77.02 ± 8.52 MPa, 25.10 ± 1.76 MPa, and 8.58 ± 0.71 MPa, respectively. Such values reflect the stiffness of the materials, in which AELO exhibits much greater stiffness than AESO and their blend. Tensile strength follows the same trend, with AELO reaching 14.94 ± 1.28 MPa, the (AELO):(AESO) blend reaching 4.39 ± 0.26 MPa, and AESO reaching 2.21 ± 0.33 MPa, further confirming that the higher degree of unsaturation of LO is directly responsible for greater mechanical properties at room temperature. It is worth noting that the strain at break for the (AELO):(AESO) blend is lower than those for the pure AELO and AESO resins. This could be indirectly related to a potential slight decreased compatibility between AELO and AESO monomers, resulting in samples that fracture prematurely during the test. This specific aspect falls outside the initial scope established for the work presented herein and will be further investigated in the future.

The results obtained for compression testing (Table 4 and Figure 10) were similar to those obtained from the tensile testing, with the AELO resin having the highest compressive modulus (722.92 ± 86.36 MPa) and strength (51.60 ± 5.37 MPa), followed by the (AELO):(AESO) blend with a compressive modulus of 681.71 ± 27.03 MPa and compressive strength of 41.95 ± 1.48 MPa, and AESO having the lowest compressive modulus (102.05 ± 3.75 MPa) and strength (9.80 ± 0.28 MPa). It is interesting to note that under tension, the (AELO):(AESO) blend exhibits a behavior closer to the AESO resin, while under compression, it behaves closer to the AELO resin. This indicates that under tension, the presence of the less reactive AESO in the blend partially works as a plasticizer, decreasing the material’s tensile properties. Under compression, the higher cross-link density imparted by the presence of AELO prevails over the plasticizing effect of AESO, increasing the material’s compressive properties. In order to confirm the initial trends observed for compression testing, a blend containing 75 wt % AELO and 25 wt % AESO was specially prepared and tested. The results indeed confirm the initial trend, with compressive strength increasing with C=C content, while compressive modulus displays a value statistically comparable to that of pure AELO when taking into account the reported standard deviations.

**Figure 10:**
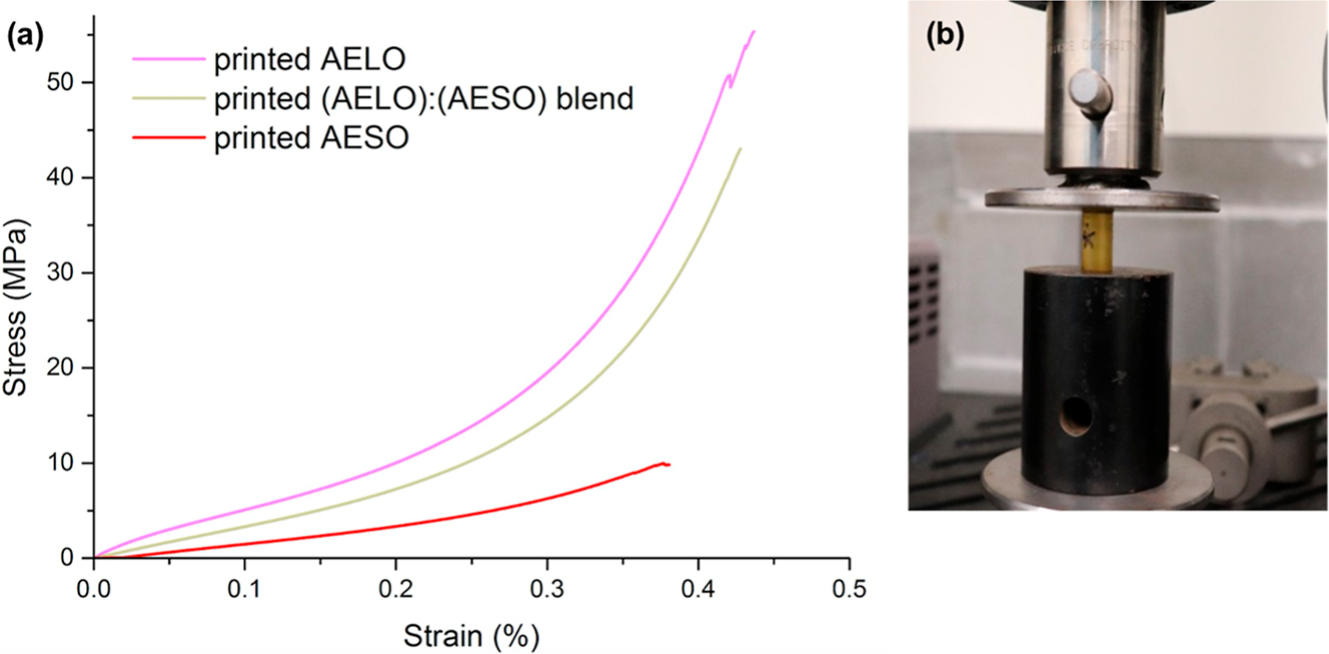
(a) Compression stress–strain curves of AESO, (AELO):(AESO) blend, and AELO. (b) Specimen under compression testing.

The hardness values (Table 4) further substantiate the conclusions drawn from tensile and compression testing results. The hardness of AELO is 57.3 ± 1.52 in the 0–100 scale, which means the material’s surface exhibit a higher resistance to deformation than the (AELO):(AESO) blend, which displays a surface hardness of 42.7 ± 0.29. That number is intermediate between AELO and AESO (hardness of 22.8 ± 0.76). This trend follows closely the trends obtained during tensile and compression testing. Furthermore, the hardness for the blend, is closer to that of AELO than from AESO, similarly to the results from compression testing. It can be therefore concluded that, just like during compression testing, the higher cross-link density imparted by the presence of AELO prevails over the plasticizing effect of AESO, increasing the material’s surface resistance to deformation. Overall, printed AESO was softer and more flexible than printed AELO and the (AELO):(AESO) blend. As previously discussed, the mechanical performance differences between these materials relate to the degree of unsaturation of the vegetable oils used in their synthesis. A higher number of carbon–carbon double bonds in unsaturated oils leads to higher mechanical properties.

## 4 Conclusions

The development and characterization of vegetable oil-based resins for 3D printing via SLA has resulted in systems that can have their properties tailored based on the blending of individual resins prepared from vegetable oils with different degrees of unsaturation. The properties of final printed parts can be altered in a predictable way by mixing oils with distinct degrees of unsaturation, which is demonstrated in this work with AESO, AELO, and blends of the two. The conversion of carbon–carbon double bonds into acrylate groups was calculated based on the ^1^H NMR results and is reported to be exactly 53% for AELO and 56% for AESO. These conversion yields are in line with other reports in the literature, being a characteristic limitation of vegetable oils.

It has been demonstrated herein that a resin prepared from AELO results in printed parts with better hardness, tensile, and compression properties than its counterpart prepared from soybean oil (AESO). Based on the mechanical properties obtained for both resins (AELO and AESO), it is clear that the higher degree of unsaturation of LO results in higher cross-link density and higher measured properties in comparison to SO. More specifically, for DMA, the storage modulus at 25 °C of AELO is over 10-fold that of AESO. Likewise, similar trends are observed for the tensile and compressive testing results. The differences in the mechanical properties of printed AELO and AESO are attributed to the different degrees of unsaturation of the oils, which is the only variable changing between the samples investigated. The different degree of unsaturation of the oils translates into a different acrylate content. The mechanical properties of the semirigid materials obtained are compatible with products that require moderate stiffness and strength, such as gaskets, seals, or dental aligners, for example.

Blends of these two resins result in printed parts with intermediary properties, with compression properties and hardness being dominated by cross-link density and therefore being closer to the properties of pure AELO, while tensile properties are dominated by AESO and its plasticizing effect. DMA data shows that the viscoelastic properties of the 50:50 (AELO):(AESO) blend are impacted by the reactivity of AELO and AESO. This is especially important at the rubbery plateau, where the polymerization kinetics of AELO and AESO impact the final cross-link density. In this work, AELO displays a lower cross-link density and *E*′ at *T*_g_ + 50 °C than the (AELO):(AESO) blend.

The choice between AELO, AESO, or a mixture of the two can be made as a function of the desired properties. This means that, since AELO has higher mechanical and thermo-mechanical properties, it would do well for applications where high strength and stiffness are required (such as structural parts), while the lower values of AESO should be better suited for applications where flexibility is desired, like rubbery mats, paddings, or dental aligners. Blends of AESO and AELO can offer intermediary values, allowing for formulations that match specific desired properties. Additionally, the cost/availability of different oils that can be possibly blended to achieve desired properties in different regions may be an important factor in deciding the optimal formulation for a certain application. The system presented herein is sufficiently versatile to provide ample room for adaptations based on needs. In summary, the degree of unsaturation and resultant polymer network developed during copolymerization of acrylated, epoxidized vegetable oils greatly influence the mechanical and thermo-mechanical properties of printed AELO, AESO, or their blend. A more detailed study using other blend proportions can be conducted in the future to provide specific compositions necessary to achieve desired properties. Future studies will also include DMA measurements in tensile mode and contrast them with flexural measurements to determine if oil functionality has a stronger effect on a specific mode of deformation. It is also worth noting that the investigation of the effect of reactive diluents and UV absorbers in the acrylated resins reported herein will be conducted in the near future. The addition of reactive diluents and UV absorbers can potentially speed up the cure, reduce printing time, and improve printing resolution and final properties of printed parts.
